# Editorial: Role of extracellular vesicles (EVs) in pathogenesis, diagnosis, therapeutic delivery, treatment and theranostic applications in cancer

**DOI:** 10.3389/fbioe.2023.1288806

**Published:** 2023-09-15

**Authors:** Reza Shahbazi, Kalimuthu Kalishwaralal, Manash K. Paul, Ruby John Anto

**Affiliations:** ^1^ Division of Hematology/Oncology, Department of Medicine, Indiana University School of Medicine, Indianapolis, IN, United States; ^2^ Indiana University Melvin and Bren Simon Comprehensive Cancer Center, Indianapolis, IN, United States; ^3^ Brown Center for Immunotherapy, Indiana University School of Medicine, Indianapolis, IN, United States; ^4^ Rajiv Gandhi Centre for Biotechnology, Thiruvananthapuram, India; ^5^ Department of Pulmonary and Critical Care Medicine, David Geffen School of Medicine, University of California Los Angeles, Los Angeles, CA, United States; ^6^ Department of Microbiology, Kasturba Medical College, Manipal Academy of Higher Education, Manipal, Karnataka, India

**Keywords:** extracellular vesicles, exosomes, cancer, proteomics, plant exosome, bacterial exosome

**FIGURE 1:**
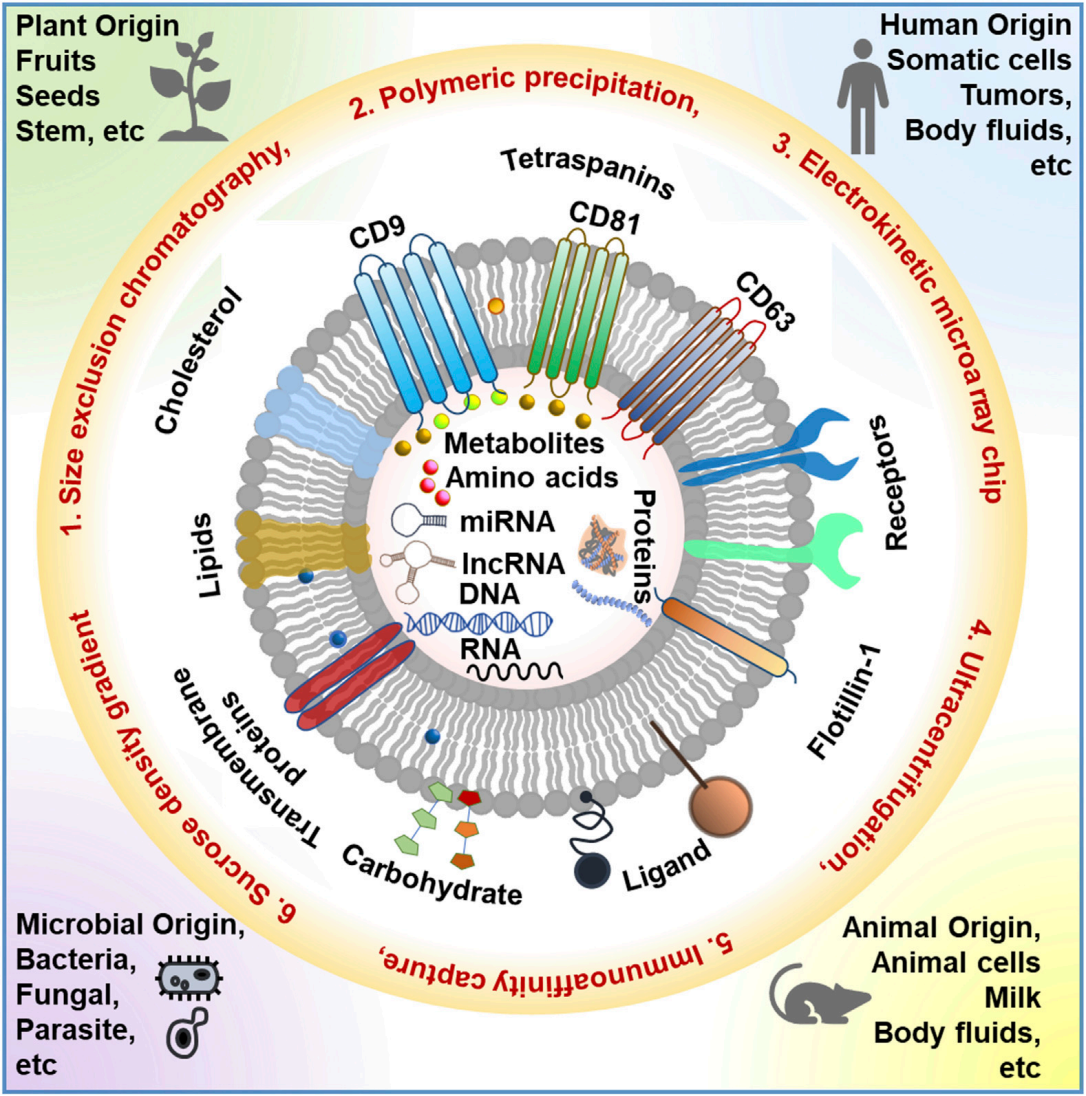
Schematic representation of the different sources of EVs, various EV isolation techniques, and EV composition. This image is inspired by DOI: 10.5772/intechopen.103865.

Extracellular vesicles (EVs) are nanoscale membranous structures that play pivotal roles in intercellular communication across various biological contexts, encompassing both health and disease. These lipid bilayer-delimited nanovesicles facilitate the horizontal transfer of biomolecular cargo, including nucleic acids, proteins, lipids, and metabolites, from donor to recipient cells, thereby establishing critical cell-to-cell communication and influencing pathological processes (Figure 1). In the context of physiological health, exosomes are indispensable for maintaining homeostasis and regulating cellular functions. They mediate the exchange of information and biomolecules between cells, contributing to processes such as immune response modulation and tissue regeneration. In cancer, exosomes assume a complex role, often promoting disease progression by transporting oncogenic cargo and modulating the tumor microenvironment. In a distinct context, plant-derived exosomes, also known as plant extracellular vesicles, are emerging as intriguing entities with potential applications in agriculture, nutrition, and biomedicine. These plant exosomes harbor bioactive compounds and genetic material, serving as mediators of communication between plants and other organisms, thereby influencing crop health and defense mechanisms. Understanding the intricate functions and diverse roles of exosomes in these contexts holds promising prospects for advancing disease diagnostics and therapeutics and revolutionizing agricultural practices.

EVs can be categorized into three principal subtypes based on their size and biological origins: exosomes (30–150 nm), microvesicles (100–1,000 nm), and apoptotic bodies (1,000–5,000 nm). Notably, in the context of cancer, tumor-derived EVs (TDEVs) are prolifically released into bodily fluids, exerting multifaceted roles in tumorigenesis. TDEVs transport tumor-promoting signaling cues, leading to the reprogramming of the tumor microenvironment (TME). These cues stimulate immunosuppression, establish metastatic niches, enhance resistance to antitumor therapies, induce metabolic alterations in other tumor cells, reprogram endothelial cells, and promote angiogenesis. Furthermore, EVs have emerged as a promising next-generation platform for antitumor therapeutics, offering several advantages over conventional delivery systems. Leveraging the principles of existing knowledge from artificial lipid-based delivery systems like liposomes, EV-based research can capitalize on their natural nanoscopic lipid-based nanocarrier properties. Moreover, endogenously derived EVs possess unique advantages in overcoming therapeutic delivery-related challenges for precise tumor targeting, including low immunogenicity, the ability to traverse the blood-brain barrier, high target specificity through surface receptor-ligand interactions, and biocompatibility. However, numerous challenges persist in scaling up EV production, developing standardized characterization protocols, minimizing batch-to-batch variations, extending shelf life, implementing novel EV bioengineering techniques for target specificity, formulating regulatory guidelines, and achieving efficient clinical translation.

This Research Topic collection serves as a comprehensive resource, offering recent insights into the roles of EVs in tumorigenesis, advancements in EV isolation techniques, applications of EVs in therapeutic delivery and proteomics, and discussions on potential sources of therapeutic EVs.

Benayas et al. have investigated the development of a high-yield and rapid EV isolation technique based on affinity separation, employing a bradykinin-derived membrane-sensing peptide (MSP). They employed agarose beads with cation chelates to precisely bind to the 6His-tagged membrane-sensing peptide, standardizing the isolation protocol for versatile EV applications.

Hadizadeh et al. conducted a comprehensive exploration of exosome biogenesis and its critical role as a biomarker in metabolic disorders. They also delved into cutting-edge technologies for exosome detection and isolation, highlighting the growing recognition of small extracellular vesicles (EVs) as versatile entities that can be harnessed for precise targeting of cellular signaling pathways, thereby offering therapeutic potential for mitigating pathological conditions. Additionally, the inherent vehicle-like properties of exosomes position them as advantageous platforms for drug and gene delivery due to their low immunogenicity.

Hou et al. have shedded light on the pivotal role of exosome in shaping the physiological processes and microenvironment of melanoma through intricate cell-to-cell communication networks. These small vesicles hold immense promise as innovative vehicles for drug delivery in melanoma treatment, especially when coupled with advanced bioengineering strategies such as surface modification and diverse loading approaches. Their review systematically categorizes advancements in exosome-based therapies for melanoma, spanning a spectrum of drug categories including chemotherapy, immunotherapy, photothermal therapy, and radiotherapy. However, they also address the substantial challenges in translating exosome-based therapies into clinical practice, including the absence of standardized isolation methods, storage protocols, and safety concerns associated with exosome-based nanomedicines. Overcoming these hurdles promises to unlock the full therapeutic potential of exosome-based strategies for melanoma in the future.

Yao et al. conducted a study comparing different isolation methods for extracellular vesicles (EVs) derived from pleural effusion (PE) in lung cancer patients. They evaluated three techniques: ultracentrifugation (UC), a combination of UC and size exclusion chromatography (UC-SEC), and a combination of UC and density gradient ultracentrifugation (UC-DGU). Their results demonstrated that the UC-SEC method exhibited the highest purity when isolating pEVs. Moreover, proteomic analysis revealed that UC-SEC isolated a greater number of proteins from pEVs compared to UC and UC-DGU. Notably, they identified novel protein markers (CD11C, HLA DPA1, and HLA DRB1) enriched in pEVs, offering valuable insights for studying diseases associated with pleural effusion.

In their review, Zhu et al. delved into the evolving role of Plant-Derived Extracellular Vesicles (PDEVs) as a highly promising nano-delivery system in the context of tumor treatment. They highlight the substantial potential of PDEVs for transporting a diverse range of cargoes, including nucleic acids, proteins, and chemotherapeutic agents, both in laboratory settings and clinical cancer treatment scenarios. However, the progress of PDEVs as a drug delivery platform, faces formidable challenges, including the lack of standardized isolation and purification methods and the timeconsuming nature of current techniques, which yield limited quantities. Moreover, a deeper understanding of PDEVs’ biological properties and transport mechanisms is imperative, including the identification of characteristic markers and surface proteins. They conclude that robust pre-clinical and large-scale clinical studies are essential to ensure the safety and efficacy of PDEVs in large-scale production. Efforts to enhance drug loading efficiency, establish optimal storage conditions, and extend *in vivo* circulation time also warrant attention.

We firmly believe that the articles featured in this Research Topic make significant contributions to our understanding of the diagnostic and therapeutic potential of EVs. These contributions equip our readers with invaluable insights, inspire innovative ideas, and foster a resolute determination to advance in this direction. Further investigations remain imperative to refine EV-based protocols, ensuring their optimal efficiency and efficacy in therapeutic delivery.

